# Case Report: Extremely elevated ascitic fluid amylase as a diagnostic clue for peritoneal mucormycosis caused by *Rhizopus microsporus* following exertional heatstroke

**DOI:** 10.3389/fmed.2026.1946492

**Published:** 2026-09-03

**Authors:** Guanghui Chen, Haibo Lei, Guyi Wang, Yanyan Zhou

**Affiliations:** 1 Department of Clinical Pharmacy, The Central Hospital of Xiangtan (The Affiliated Hospital of Hunan University), Xiangtan, China; 2 Department of Critical Care Medicine, The Second Xiangya Hospital, Central South University, Changsha, China

**Keywords:** ascitic fluid amylase, heatstroke, peritoneal mucormycosis, *Rhizopus microsporus*, targeted next-generation sequencing

## Abstract

**Background:**

Mucormycosis is a rapidly progressive, life-threatening fungal infection that typically affects immunocompromised hosts. Disseminated peritoneal mucormycosis is extremely rare and often diagnosed late. Heatstroke is an under-recognized predisposing condition.

**Case presentation:**

A 54-year-old male brickworker developed peritoneal mucormycosis caused by *Rhizopus microsporus* following exertional heatstroke. On day 11 after heatstroke, he developed massive ascites. Ascitic fluid amylase was markedly elevated to 51,808 U/L, while plasma amylase remained low (40.3 U/L). Contrast study excluded perforation, and CT showed a normal pancreas. Sputum fungal culture grew *Rhizopus* species on day 4. Targeted next-generation sequencing (tNGS) of ascitic fluid and blood identified *R. microsporus* (151 and 53 reads). Tissue biopsy was precluded by severe coagulopathy and thrombocytopenia. The patient received liposomal amphotericin B (L-AmB) plus isavuconazole; subsequent detection of *Candida tropicalis* superinfection prompted a switch to caspofungin while continuing L-AmB. Ascitic fluid amylase declined progressively to 38 U/L, with radiological resolution. The patient recovered without surgical debridement.

**Conclusion:**

Extremely elevated ascitic fluid amylase with a normal pancreas and excluded perforation should prompt consideration of peritoneal mucormycosis. This case suggests that fungal-derived enzymes may contribute to the enzyme elevation, though this remains speculative without direct assays. Combination antifungal therapy achieved cure despite contraindications to surgery. Heatstroke is an important but under-recognized risk factor for disseminated mucormycosis.

## Introduction

Mucormycosis is a rare but highly lethal opportunistic infection caused by *Mucorales*, with mortality exceeding 40% in disseminated disease (1). Classical risk factors include hematological malignancies, diabetes mellitus, and immunosuppressive therapy; yet 10–19% of cases arise in patients lacking these traditional predisposing conditions (1–3). Heatstroke, a syndrome characterized by extreme hyperthermia and multiorgan failure, compromises gut barrier integrity through splanchnic ischemia and a dysregulated systemic inflammatory response (4). While heatstroke-associated mucormycosis has been reported in a single case of isolated gastric involvement (5), disseminated peritoneal mucormycosis with hematogenous spread following heatstroke has not, to our knowledge, been documented. This case report describes a patient with exertional heatstroke who developed peritoneal mucormycosis caused by *R. microsporus*, highlighting both a novel diagnostic clue, extremely elevated ascitic fluid amylase, and the successful use of combination antifungal therapy without surgical intervention.

## Case presentation

**FIGURE 1:**
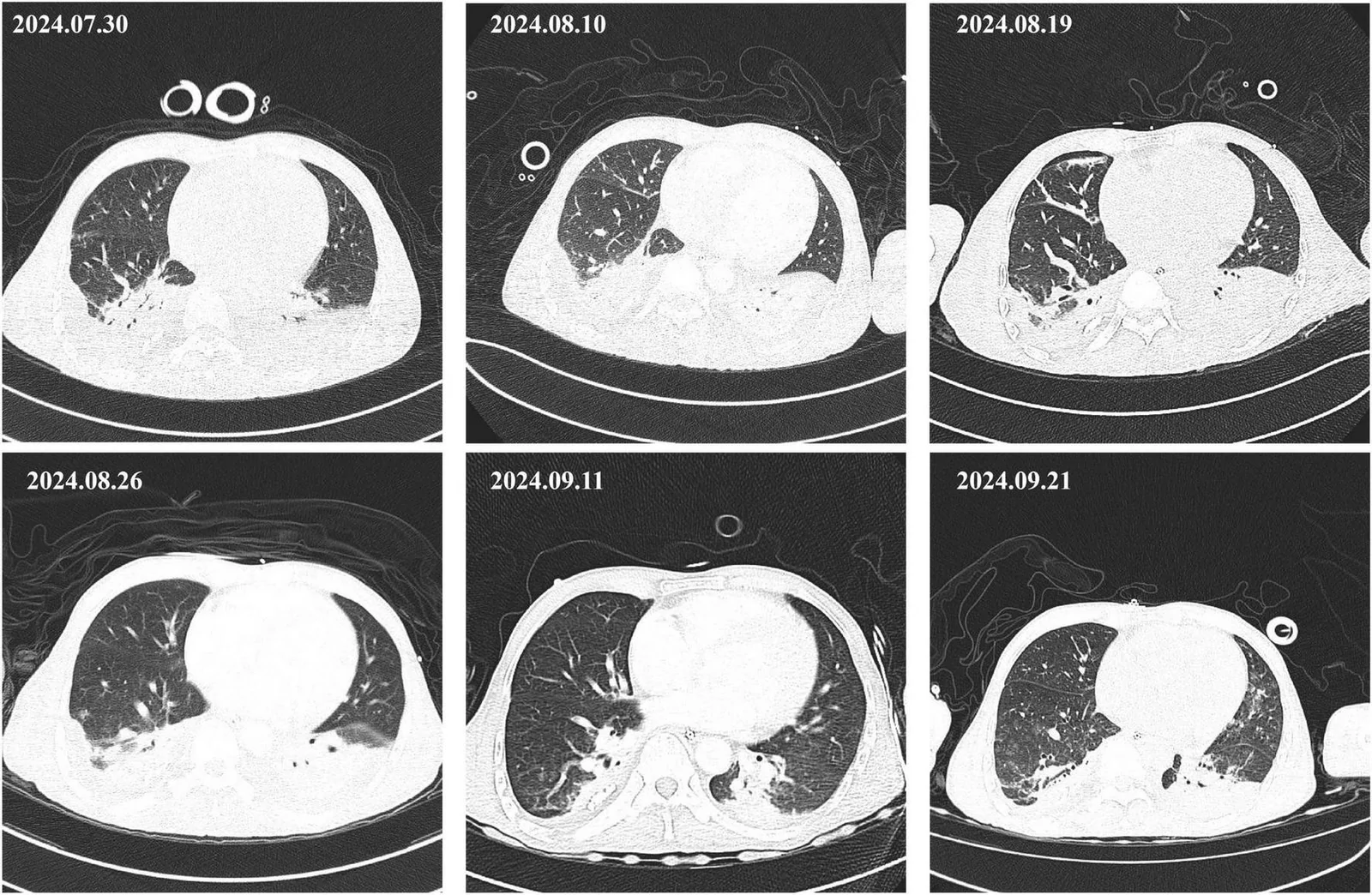
Serial chest CT images demonstrate gradual resolution of bilateral pulmonary infiltrates and pleural effusion over the 65-day hospitalization.

A 54-year-old male brickworker with no history of diabetes, malignancy, or immunosuppression was transferred to our intensive care unit on 27 July 2024 with a 4-day history of progressively impaired consciousness after prolonged manual labor in extreme heat (ambient temperature approximately 39 °C). The patient had heavy occupational exposure to dust as a brickworker, which likely facilitated inhalation of fungal spores. His core temperature at the scene was reportedly 41.2 °C. On admission, he was comatose (Glasgow Coma Scale score 7), with a recorded temperature of 37.8 °C (having received external cooling during transfer), blood pressure 114/70 mmHg on noradrenaline 0.3 μg/kg/min, and laboratory evidence of multiorgan failure: aspartate aminotransferase 5299.6 U/L, alanine aminotransferase 3346.8 U/L, serum creatinine 162 μmol/L, international normalized ratio 2.64, and interleukin-6 > 5000 pg/mL. Serum amylase and lipase were normal (69 U/L and 49.3 U/L). Computed tomography (CT) on admission showed massive ascites and bilateral pulmonary infiltrates (Figure 1). He was immediately managed with active cooling, continuous renal replacement therapy (CRRT), invasive mechanical ventilation, and empirical meropenem.

**FIGURE 2:**
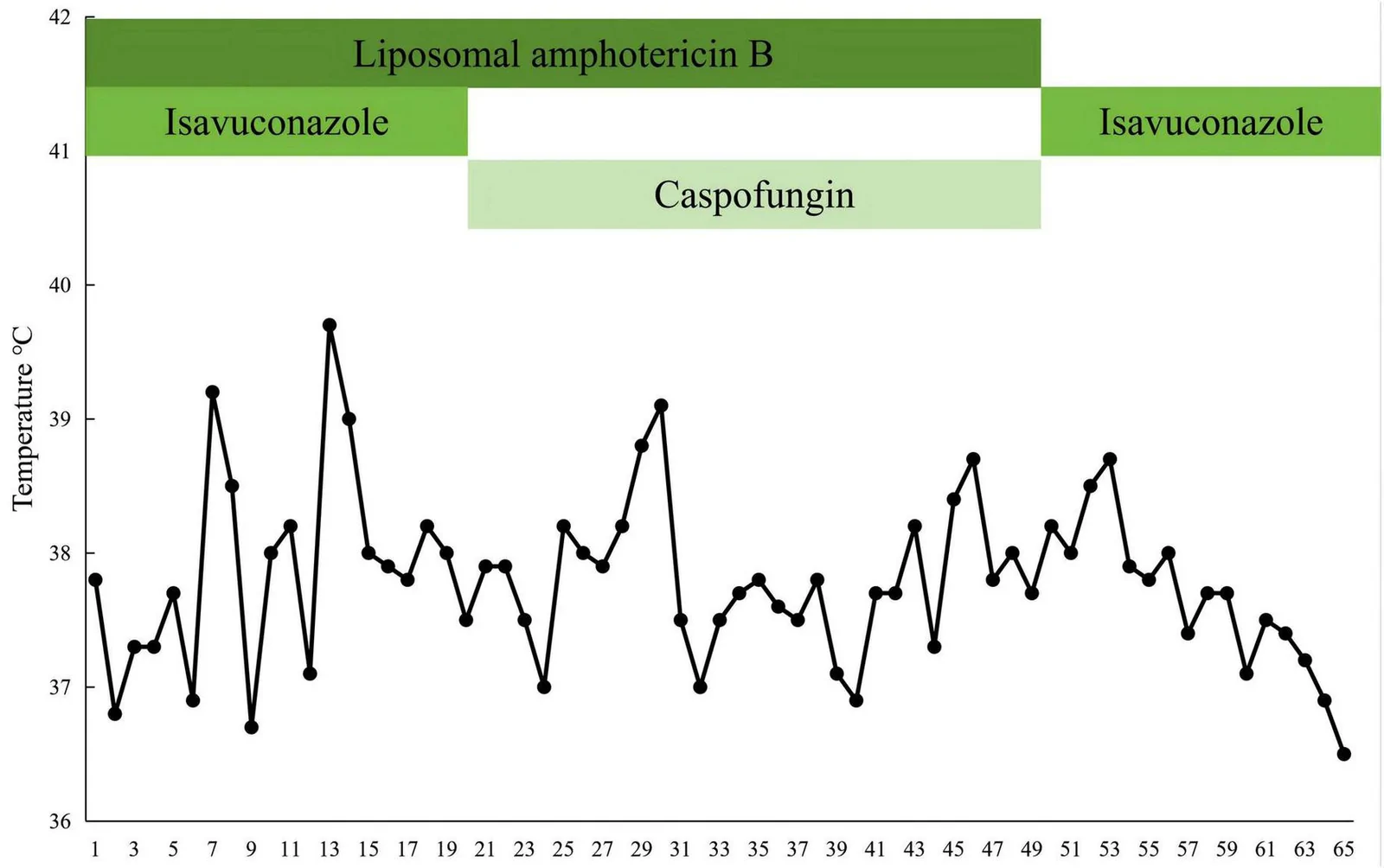
Changes in temperature and antifungal use of the patient during hospitalization.

On day 3, targeted next-generation sequencing (tNGS) of bronchoalveolar lavage fluid (BALF) detected *R. microsporus* (1090 reads). On day 4, sputum fungal culture provided definitive microbiological confirmation by growing *Rhizopus* species, independently corroborating the pulmonary involvement. Combination antifungal therapy was initiated immediately with liposomal amphotericin B (L-AmB) 350 mg/day (5 mg/kg/day) plus intravenous isavuconazole (200 mg every 8 h for six doses, then 200 mg/day). The patient’s temperature course and antifungal timeline are summarized in Figure 2.

**FIGURE 3:**
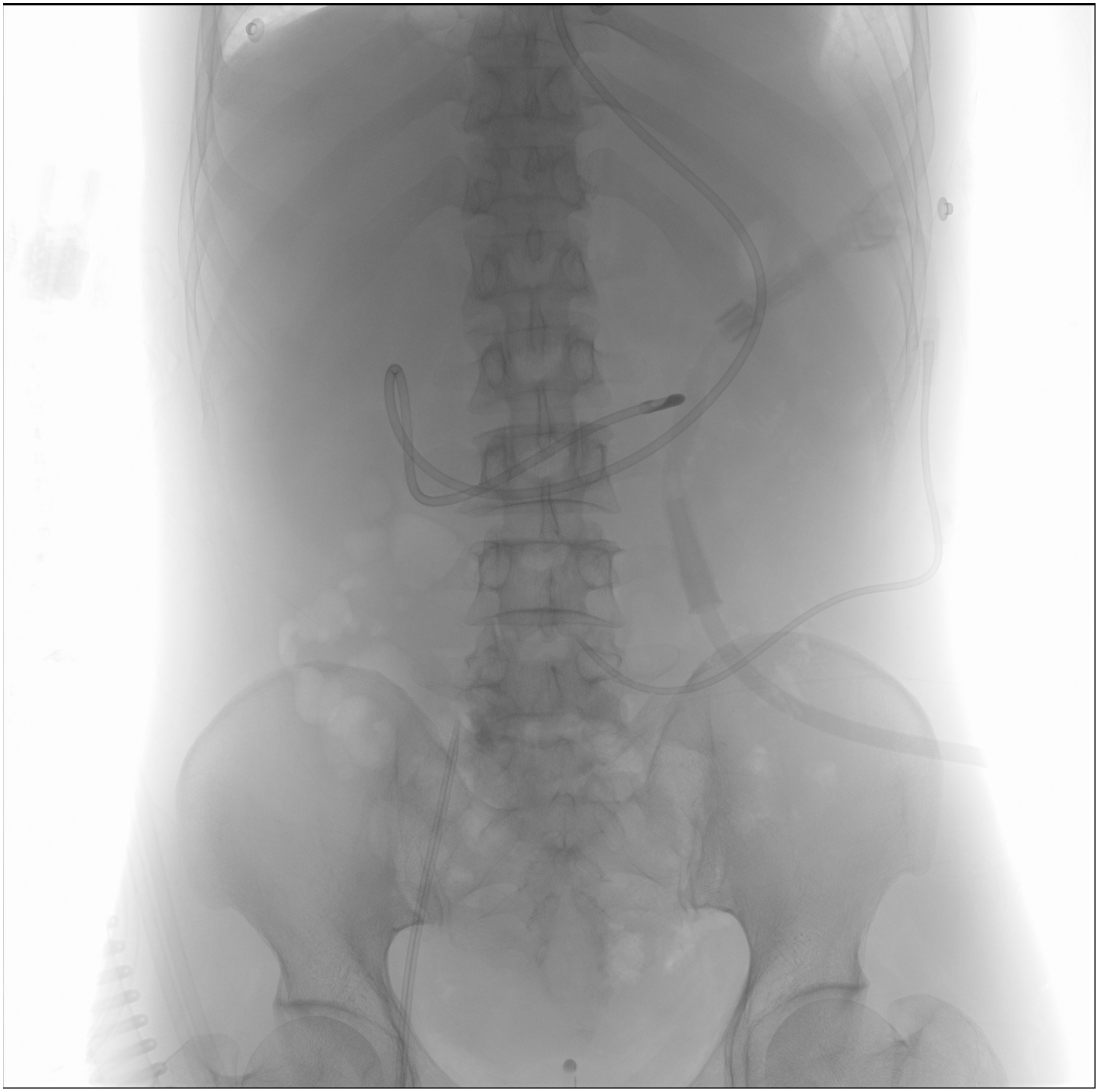
Water-soluble contrast enema shows a gas-containing cystic lesion inseparable from the distal ileum, without free contrast extravasation or intraperitoneal air.

**FIGURE 4:**
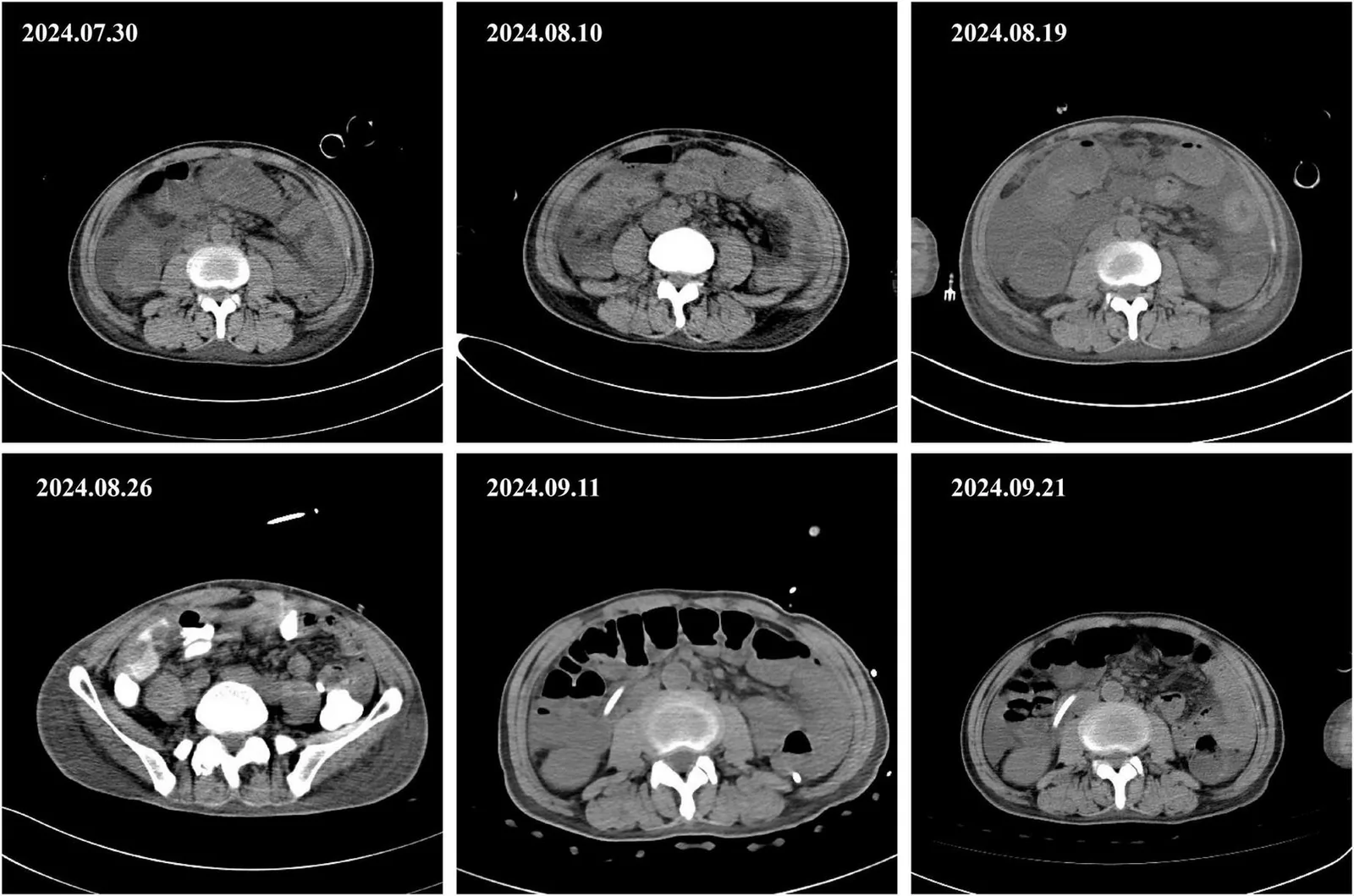
Serial abdominal CT images show evolution from massive ascites to a right lower quadrant cystic lesion containing a small amount of gas, with complete resolution of both by day 65, paralleling the decline of ascitic fluid amylase and fungal clearance.

On day 11, the patient developed progressive abdominal distension with massive ascites. Diagnostic paracentesis showed peak ascitic fluid amylase (51,808 U/L) and lipase (2,405.1 U/L), while plasma amylase remained low (40.3 U/L on day 11, Table 2). tNGS of ascitic fluid and blood identified *R. microsporus* (151 and 53 reads). To exclude bowel perforation, an urgent water-soluble contrast enema with CT was performed. No free extraluminal contrast or intraperitoneal air was observed, effectively ruling out a free perforation (Figure 3). Contrast-enhanced CT confirmed a completely normal pancreas. Instead, a thick-walled cystic lesion containing a small amount of gas was identified in the right lower quadrant, abutting a loop of distal ileum (Figure 4). A sinus tract connecting the lesion to the bowel lumen was suspected, consistent with transmural necrosis without free perforation. Fungal culture of ascitic fluid was negative, and tissue biopsy was not performed due to severe coagulopathy and thrombocytopenia (platelet nadir 24 × 10^9^/L).

**TABLE 1:** The tNGS results of the patient.

Number	Sample	Date	Pathogen detected	Reads (*n*)
1	BALF	2024/7/29	*R. microsporus*	1090
2	Ascitic fluid	2024/8/6	*R. microsporus*	151
3	Plasma	2024/8/6	*R. microsporus*	53
4	Ascitic fluid	2024/8/14	*Candida tropicalis*	42052
5	Plasma	2024/8/14	ND	ND
6	Ascitic fluid	2024/9/11	ND	ND
7	Plasma	2024/9/12	ND	ND

BALF, bronchoalveolar lavage fluid; ND, Not Detected. Only clinically significant fungal pathogens are shown.

**TABLE 2:** Serial ascitic fluid and plasma amylase and lipase levels.

Number	Date (2024)	Sample	Amylase (U/L)	Lipase (U/L)
1	29 Jul	Plasma	32	19
2	31 Jul	Ascitic fluid	49.3	28
3	2 Aug	Ascitic fluid	136.9	40.3
4	3 Aug	Plasma	40.3	39
5	5 Aug	Ascitic fluid	15,990	820
6	6 Aug	Ascitic fluid	51,808	2405.1
7	10 Aug	Ascitic fluid	16820	2252.1
8	12 Aug	Plasma	183.4	102
9	12 Aug	Ascitic fluid	17915	2405.1
10	14 Aug	Ascitic fluid	579	2288.6
11	15 Aug	Ascitic fluid	376	2252.1
12	15 Aug	Plasma	109	217.1
13	16 Aug	Ascitic fluid	299	1798.5
14	17 Aug	Ascitic fluid	496	1522.5
15	20 Aug	Ascitic fluid	99	956.6
16	25 Aug	Ascitic fluid	71	87.5
17	4 Sep	Ascitic fluid	59	63
18	10 Sep	Ascitic fluid	45	52.8
19	19 Sep	Ascitic fluid	38	41

On day 19 (14 August 2024), repeat ascitic fluid tNGS revealed a superinfection with *Candida tropicalis* (42,052 reads). Isavuconazole was therefore replaced by caspofungin (50 mg/day after a 70 mg loading dose), while L-AmB was continued.

Over the following weeks, the patient’s abdominal distension and ascites gradually resolved. Serial ascitic fluid amylase and lipase declined to 38 U/L and 41 U/L by day 55 (Tables 2, 3). Ascitic fluid analysis (Table 3) evolved from an initial exudative pattern (WBC 5,250/μL, LDH 1,299 U/L on day 7) to a transudative pattern (WBC 62/μL, LDH 191 U/L on day 46), consistent with resolving peritonitis. Follow-up CT on day 42 demonstrated complete resolution of the cystic lesion and ascites. Serial tNGS subsequently became negative for *Rhizopus*. On day 50, therapy was de-escalated to oral isavuconazole monotherapy. The patient’s consciousness and organ function gradually recovered, and he was discharged on day 65.

**TABLE 3:** Serial ascitic fluid routine and biochemical parameters.

Date (2024)	Color	Clarity	Rivalta test	WBC count (10^6/L))	PMN (%)	LDH (U/L)	ADA (U/L)	Ascitic albumin (g/L)	Serum albumin (g/L)
Aug 2	Yellow	Turbid	Positive (3+)	5250	80	1298.8	12.3	19.9	29.9
Aug 6	Yellow	Turbid	Positive (2+)	800	60	276.9	7.0	20.7	33.6
Aug 9	Light red	Turbid	Positive (3+)	1750	40	642.0	17.0	23.9	36.5
Aug 14	Yellow	Turbid	Positive (2+)	980	30	503.3	7.7	21.3	35.9
Sep 10	Dark yellow	Clear	Positive (1+)	62	35.5	191.0	5.1	22.7	35.6
Sep 19	Yellow	Turbid	Positive (1+)	7	28.6	141.6	12.1	25.6	31.1

PMN, polymorphonuclear cells (including neutrophils). On Sep 10, PMN count was 22 out of 62 WBCs, corresponding to 35.5%. On Sep 19, PMN count was 2 out of 7 WBCs, corresponding to 28.6%.

## Discussion

This case provides the first detailed description of peritoneal mucormycosis caused by *R. microsporus* following exertional heatstroke, with tNGS detection of fungal DNA in both ascitic fluid and blood. Mucormycosis carries a mortality rate of 40–60% in disseminated disease, rising to 85% when gastrointestinal involvement occurs (6, 7). Although typically linked to hematological malignancies and diabetes, 10–19% of cases occur in patients without classical risk factors (2). The most striking finding, ascitic fluid amylase reaching 51,808 U/L in the absence of acute pancreatitis or bowel perforation, offers a novel diagnostic clue. A previous case of gastric mucormycosis following heatstroke was reported (5), but without peritoneal dissemination. Most published cases of peritoneal mucormycosis have occurred in immunocompromised patients (6, 7), whereas our patient’s only identifiable risk factor was heatstroke-induced immunoparalysis and gut barrier disruption. Serial peripheral blood white blood cell counts and differentials during hospitalization are presented in Supplementary Table 1.

Prolonged heat exposure triggers a massive systemic inflammatory response that, when sustained, shifts the immune landscape toward compensatory immune paralysis (4, 8). Mechanistically, heat stress upregulates anti-inflammatory cytokines (IL-10, TGF-β) and suppresses TLR4/NF-κB signaling in monocytes and neutrophils, impairing phagocytosis and oxidative burst (8, 9). This transient immunoparalysis compromises innate defenses against filamentous fungi. Simultaneously, heat-induced splanchnic hypoperfusion disrupts intestinal epithelial tight junctions, facilitating microbial translocation (10). In our patient, heavy dust exposure likely led to pulmonary inoculation of *R. microsporus* spores; subsequent immunoparalysis permitted hematogenous dissemination, as suggested by sequential tNGS (BALF→blood→ascitic fluid).

*R. microsporus* is a prolific producer of amylolytic and lipolytic enzymes in industrial biotechnology (11, 12). The optimal activity of *Rhizopus* lipases at 30–45°C and pH 6.0–8.5, together with their stability at 37°C (11), suggests that secreted enzymes could remain active in ascitic fluid and contribute to local lipolysis and amylolysis. Metaproteomic analyses of Chinese liquor fermentation have identified it as the dominant saccharifying fungus, contributing > 60% of total microbial proteins and serving as the principal source of glucoamylase, which remains fully stable at 37°C (12). Crucially, recent studies have confirmed such activity *in vivo*: a seminal study identified *Rhizopus*-specific glucoamylase (RSG) as an immunodominant antigen secreted by *R. arrhizus* during infection, eliciting robust anti-RSG IgG responses in mucormycosis patients (13). Moreover, Nordén et al. reported a case of wound zygomycosis caused by a lipase-producing *R. rhizopodiformis* strain, with histopathological evidence of fatty necrosis and fatty acid crystallization in infected tissue, indicating *in vivo* lipolysis of host fat by fungal lipase (14).

Based on these observations, we hypothesize that the extreme hyperamylasemia and hyperlipasemia resulted in part from direct secretion by viable *R. microsporus* in the peritoneal cavity, with angioinvasive necrosis providing the release pathway. Supporting this, pancreatitis and perforation were excluded, and both enzymes declined with antifungal therapy. However, alternative explanations cannot be excluded. A contained perforation or enteric fistula could also cause enzyme leakage, and without histopathology or direct enzyme assays, the relative contributions remain uncertain.

The diagnostic process was greatly facilitated by tNGS. Traditional culture for *Mucorales* has low sensitivity ( < 50%) and a slow turnaround (15), whereas metagenomic NGS can distinguish infection from colonization using a proposed threshold of 51 reads (16). In our case, multi-site serial tNGS (Table 1) detected *R. microsporus* at all sampled sites, with reads exceeding the threshold, providing supportive molecular evidence. Sputum culture independently confirmed the presence of *Rhizopus* species. However, definitive diagnosis requires histopathology or sterile-site culture, neither feasible here; the diagnosis therefore remains probable.

The global guideline recommends L-AmB at 5–10 mg/kg/day, with isavuconazole as an alternative or step-down option (1). High-quality evidence for combination therapy in mucormycosis remains scarce (3). A systematic review reported encouraging recovery rates with intravenous combination therapy followed by oral isavuconazole, noting mortality rates of 32% for L-AmB monotherapy, 6.6% for L-AmB plus an azole, and 24.6% for isavuconazole monotherapy (17). Although these figures should be interpreted with caution given the predominantly observational nature of the included studies, they suggest a potential benefit of combination therapy. Furthermore, a mouse model of pulmonary mucormycosis showed that L-AmB plus isavuconazole was synergistic (18). Repeat tNGS detected *Candida tropicalis*, superinfection prompting a switch to caspofungin per guidelines (19). The patient recovered without surgery, which was contraindicated due to persistent coagulopathy and thrombocytopenia. This case provides real-world evidence that combination antifungal therapy may be effective when surgical debridement is not feasible.

Limitations include the absence of histopathology, negative ascitic fluid culture, and lack of direct enzyme assays. Despite these limitations, this case highlights the diagnostic value of extremely elevated ascitic fluid amylase in suspected peritoneal mucormycosis and suggests that combination antifungal therapy may be effective when surgery is contraindicated.

## Conclusion

An extreme elevation of ascitic fluid amylase in the absence of pancreatitis or free bowel perforation should prompt consideration of peritoneal mucormycosis. This case suggests that *R. microsporus* may secrete amylase and lipase during invasive infection, though this remains speculative without direct enzymatic confirmation. Exertional heatstroke is an under-recognized predisposing condition, acting through immunoparalysis and gut barrier disruption. Multi-site serial tNGS can provide rapid molecular evidence to support diagnosis and guide therapy. Importantly, combination antifungal therapy can be curative even when surgery is contraindicated, offering real-world evidence where high-quality data are scarce.

## Data Availability

The original contributions are included in the article. Further inquiries can be directed to the corresponding author.
