# Supplementary material for: Case Report: Extremely elevated ascitic fluid amylase as a diagnostic clue for peritoneal mucormycosis caused by Rhizopus microsporus following exertional heatstroke
Source: Front Med (Lausanne). 2026 Sep 3;13:1946492. doi: 10.3389/fmed.2026.1946492 (PMC13579790; doi:10.3389/fmed.2026.1946492)
Supplement: Supplementary file 1 [file Table_1.docx]

### **Supplementary Table 1. Serial peripheral blood white blood cell counts and differentials**

| Date (2024) | WBC (×10^9^/L) | Neutrophils (%) | Lymphocytes (×10^9^/L) |
| --- | --- | --- | --- |
| 27 Jul | 4.09 | 87.6 | 0.38 |
| 28 Jul | 2.26 | 88.5 | 0.16 |
| 29 Jul | 4.26 | 85.5 | 0.21 |
| 30 Jul | 6.51 | 84.5 | 0.36 |
| 31 Jul | 8.09 | 80.4 | 0.53 |
| 1 Aug | 5.51 | 81.6 | 0.52 |
| 2 Aug | 5.41 | 81.6 | 0.49 |
| 3 Aug | 6.50 | 85.4 | 0.60 |
| 4 Aug | 6.06 | 84.7 | 0.41 |
| 5 Aug | 5.62 | 82.0 | 0.49 |
| 6 Aug | 5.62 | 79.1 | 0.65 |
| 7 Aug | 4.47 | 81.2 | 0.38 |
| 8 Aug | 6.21 | 78.6 | 0.65 |
| 9 Aug | 9.04 | 77.9 | 0.91 |
| 10 Aug | 8.92 | 86.6 | 0.58 |
| 11 Aug | 12.16 | 87.6 | 0.54 |
| 12 Aug | 14.58 | 83.6 | 0.95 |
| 13 Aug | 16.85 | 84.3 | 0.96 |
| 14 Aug | 23.91 | 88.7 | 0.68 |
| 15 Aug | 22.86 | 87.0 | 0.98 |
| 16 Aug | 19.72 | 84.3 | 1.12 |
| 17 Aug | 20.79 | 83.0 | 1.43 |
| 18 Aug | 20.67 | 84.9 | 1.07 |
| 19 Aug | 17.37 | 88.9 | 0.75 |
| 20 Aug | 18.03 | 91.0 | 0.74 |
| 21 Aug | 16.57 | 88.3 | 0.97 |
| 22 Aug | 14.97 | 87.8 | 0.79 |
| 23 Aug | 23.68 | 93.3 | 0.55 |
| 24 Aug | 11.91 | 88.5 | 0.64 |
| 25 Aug | 12.86 | 88.1 | 0.62 |
| 26 Aug | 9.54 | 84.8 | 0.50 |
| 27 Aug | 7.07 | 82.2 | 0.53 |
| 28 Aug | 6.71 | 81.2 | 0.74 |
| 29 Aug | 6.59 | 82.4 | 0.50 |
| 30 Aug | 6.79 | 83.0 | 0.61 |
| 31 Aug | 5.22 | 79.7 | 0.57 |
| 1 Sep | 4.66 | 79.9 | 0.40 |
| 3 Sep | 4.05 | 79.3 | 0.44 |
| 4 Sep | — | — | — |
| 8 Sep | 4.64 | 77.2 | 0.70 |
| 9 Sep | 3.93 | 78.6 | 0.60 |
| 10 Sep | 5.65 | 80.5 | 0.81 |
| 11 Sep | 4.94 | 81.1 | 0.69 |
| 12 Sep | 4.55 | 84.1 | 0.46 |
| 13 Sep | 3.85 | 81.6 | 0.47 |
| 14 Sep | 4.75 | 77.5 | 0.66 |
| 15 Sep | 5.25 | 72.8 | 0.81 |
| 16 Sep | 6.39 | 72.1 | 1.05 |
| 19 Sep | 9.57 | 85.8 | 0.85 |
| 20 Sep | 6.75 | 82.9 | 0.83 |
| 21 Sep | 5.99 | 79.8 | 0.79 |
| 24 Sep | 5.42 | 83.6 | 0.43 |
| 25 Sep | 4.98 | 69.9 | 1.01 |
| 27 Sep | 4.83 | 72.9 | 0.85 |
| 28 Sep | 5.09 | 75.1 | 0.80 |
